# Oncologic necessity for the complete removal of residual microcalcifications after neoadjuvant chemotherapy for breast cancer

**DOI:** 10.1038/s41598-022-24757-7

**Published:** 2022-12-13

**Authors:** Jeeyeon Lee, Nora Jee-Young Park, Ho Yong Park, Wan Wook Kim, Byeongju Kang, Heejung Keum, Hye Jung Kim, Won Hwa Kim, Yee Soo Chae, Soo Jung Lee, In Hee Lee, Ji-Young Park, Jin Hyang Jung

**Affiliations:** 1 Department of Surgery, School of Medicine, Kyungpook National University, Daegu, Republic of Korea; 2 Department of Pathology, School of Medicine, Kyungpook National University, Daegu, Republic of Korea; 3 Department of Radiology, School of Medicine, Kyungpook National University, Daegu, Republic of Korea; 4 Department of Hematology/Oncology, School of Medicine, Kyungpook National University, Daegu, Republic of Korea; 5 Kyungpook National University Chilgok Hospital, Daegu, Republic of Korea

**Keywords:** Breast cancer, Cancer, Oncology

## Abstract

The surgical range of breast cancer that shows pathologic complete response (pCR) without change in microcalcifications after neoadjuvant chemotherapy (NAC) is controversial. This study examined whole breast specimens to evaluate the necessity of mastectomy in those cases. The viability of cancer cells around the residual microcalcification was assessed using prospectively collected breast samples to confirm the presence or absence of cancer cells. A total of 144 patients with breast cancer and diffuse microcalcifications were classified into the reduced mass with no change in residual microcalcification (RESMIN, n = 49) and non-RESMIN (n = 95) groups. Five specimens were prospectively evaluated to assess the presence of viable cancer cells around the microcalcification. Tumor responses to NAC were significantly better with high pCR rates in the RESMIN group (p = 0.005 and p = 0.002). The incidence of human epidermal growth factor receptor 2-positive and triple-negative breast cancers was significantly high in the RESMIN group (p = 0.007). Although five (10.2%) patients had locoregional recurrence in the RESMIN group, no local recurrence in the breast was reported. Although pCR was highly estimated, residual cancers, including ductal carcinoma in situ, remained in 80% cases. Therefore, given the weak scientific evidence available currently, complete removal of residual microcalcifications should be considered for oncologic safety.

## Introduction

Although the most common clinical symptom of breast cancer is a palpable lump, microcalcifications are also a common clinical finding; these findings are occasionally found concurrently^1^. Microcalcifications in the breast are deposits of calcium products that can be visualized as < 1-mm bright white spots on mammography^2^. Fine, linear, or branched microcalcifications, which usually extend along the duct, are often accompanied with ductal carcinoma in situ (DCIS)^3–8^. However, these malignant microcalcifications are also frequently detected in advanced breast cancers with large palpable lumps.

In advanced breast cancer, neoadjuvant chemotherapy (NAC) is administered to convert an inoperable breast cancer to an operable one to perform breast-conserving surgery and avoid axillary lymph node dissection^9–13^. The therapeutic effect of NAC is higher in aggressive breast cancers such as human epidermal growth factor receptor 2 (HER2)-positive or triple-negative breast cancers (TNBC), leading to a significant decrease in the tumor size. Although patients with advanced breast cancer exhibit significant tumor shrinkage after receiving NAC, diffuse microcalcifications often remain, regardless of the change in the primary breast tumor.

In general, the type of surgery for breast cancer is determined according to the size of the breast tumor and range of microcalcification. The surgical scale can be easily determined when the size of breast tumor and range of microcalification match. However, there is a lack of consensus on determining the surgical scale in cases of mismatch. Even if the tumor size is reduced by NAC, performing breast-conserving surgery or reducing the surgical scale can be especially difficult when diffuse microcalcifications remain after NAC. Many studies have reported that the complete removal of residual microcalcifications after NAC for breast cancer appears to be safe^14–17^. However, malignant microcalcifications containing larger hydroxyapatite particles, even if detected as an early finding in breast cancer, are associated with more invasive breast carcinomas^18^. Because hydroxyapatite upregulates the expression of matrix metalloproteinase 1 (MMP-1) and promotes the migration of breast cancer cells through decreased elasticity of the extracellular matrix, augmented gene expression of MMP-1 predicts worse metastasis-free survival^19,20^.

Several studies have reported scientific evidence in support of mastectomy for breast cancers that show a pathologic complete response (pCR) without a change in microcalcifications after NAC. However, the clinical and pathologic evidence is weak because no study has investigated and analyzed the presence of breast cancer around residual microcalcifications. Therefore, this study aimed to establish the clinical implication of complete removal of residual microcalcifications by investigating the changes in breast cancer and microcalcification after NAC retrospectively and evaluating whole breast specimens to prove the necessity of mastectomy in those cases by evaluating viable cancer cells around residual microcalcifications. We established the clinical implication with the same indication as retrospectively.

## Results

The pCR rate was significantly higher in the reduced mass with no change in residual microcalcification (RESMIN) group (n = 19, 38.8 %) than in the non-RESMIN group (n = 19, 20.0 %) (p = 0.002). In addition, the overall tumor responses to NAC were also significantly better in the RESMIN group than in the non-RESMIN group (p = 0.005). Sentinel lymph node biopsy was performed more frequently in the RESMIN group (n = 25, 51.0 %) than in the non-RESMIN group (n = 32, 33.7 %) (p = 0.018).

Most clinicopathologic factors, including the incidence of breast reconstruction, hormone receptor (HR) status, HER2/neu gene status, and Ki67 index, showed no differences between the total and RESMIN groups. However, the incidence of HER2-positive breast cancer (RESMIN: n = 26, 53.1 % vs. non-RESMIN: n = 39, 41.1%; p = 0.041) and TNBC (RESMIN: n = 5, 10.2 % vs. non-RESMIN: n = 9, 9.5 %; p = 0.007) was significantly higher in the RESMIN group than in the non-RESMIN group (Table 1).

**Table 1:** Clinicopathologic characteristics of patients with breast cancer with microcalcification who received neoadjuvant chemotherapy followed by mastectomy.

Characteristics	RESMIN (n = 49)	Non-RESMIN (n = 95)	p-value
**Age at diagnosis (mean ± SD, years)**	46.6 ± 6.4	49.16 ± 18.4	0.467
**Pathologic complete response (pCR) (n, %)**	19 (38.8)	19 (20.0)	0.002
**Type of axillary surgery (n, %)**			0.018
Sentinel lymph node biopsy	25 (51.0)	32 (33.7)	
Axillary lymph node dissection	24 (49.0)	63 (66.3)	
Immediate breast reconstruction	10 (20.4)	18 (19.0)	0.051
**Estrogen receptor (n, %)**			0.221
Positive	33 (67.4)	63 (66.3)	
Negative	16 (32.7)	38 (40.0)	
**Progesterone receptor (n, %)**			0.920
Positive	24 (49.0)	52 (54.7)	
Negative	25 (51.0)	43 (45.3)	
**HER2 gene (n, %)**			0.041
Positive	26 (53.1)	39 (41.1)	
Negative	23 (46.9)	56 (59.0)	
Triple negative breast cancer (n, %)	5 (10.2)	9 (9.5)	0.007
**Ki67 index (n, %)**			0.145
High	34 (69.4)	60 (63.2)	
Low	15 (30.6)	35 (36.9)	
**Neoadjuvant chemotherapy (n, %)**			N/A
Adriamycin + Cyclophosphamide		6 (6.3)	
Paclitaxel only	1 (2.0)	5 (5.0)	
Adriamycin + Cyclophosphamide → Docetaxel	40 (81.6)	77 (81.1)	
Docetaxel → 5-FU + Epirubicin + Cyclophosphamide	3 (6.13)	2 (2.1)	
Docetaxel + Carboplatin + Trastuzumab + Pertuzumab	0	1 (1.1)	
Paclitaxel + Carboplatin + /−Velaparib	3 (6.13)	0	
Doxitaxel + Carboplatin	1 (2.0)	4 (4.2)	
Paclitaxel + Carboplatin	1 (2.0)	0	
**Tumor response in neoadjuvant chemotherapy (n, %)**			0.005
Complete response	26 (53.1)	14 (14.7)	
Partial response	23 (46.9)	74 (77.9)	
Stable disease (no response)	0	4 (4.2)	
Progressive disease	0	3 (3.2)	
**Adjuvant chemotherapy (n, %)**	0	2 (2.1)	0.326
**Target therapy (n, %)**	26 (53.1)	42 (44.2)	0.612
**Adjuvant radiotherapy (n, %)**	44 (89.8)	81 (85.3)	0.262
**Adjuvant hormonal therapy (n, %)**	34 (69.4)	65 (68.4)	0.079

In both groups, the mean tumor size on ultrasonography (RESMIN: 1.9 ± 0.3 cm vs. non-RESMIN: 3.2 ± 1.6 cm) and breast magnetic resonance imaging (RESMIN: 1.3 ± 0.3 cm vs. non-RESMIN: 2.7 ± 0.9 cm) after NAC was greatly reduced compared to that at the initial diagnosis before NAC. However, the extent of microcalcification on mammography did not change as much in both groups (RESMIN: 5.1 ± 0.4 cm vs. non-RESMIN: 4.8 ± 0.7 cm) (Table 2, Supplementary Fig. 1).

**Table 2:** Tumor characteristics of patients with breast cancer with microcalcification who received neoadjuvant chemotherapy followed by mastectomy.

	RESMIN (n = 49)		Non-RESMIN (n = 95)	
	Pre-NAC	Post-NAC	Pre-NAC	Post-NAC
**Tumor extent (mean ± SD, cm)**				
In mammography including microcalcification	5.4 ± 0.7	5.1 ± 0.4	5.2 ± 0.5	4.8 ± 0.7
In ultrasonography	5.2 ± 0.7	1.9 ± 0.3	5.0 ± 0.7	3.2 ± 1.6
In breast MR	5.8 ± 1.1	1.3 ± 0.3	5.6 ± 2.5	2.7 ± 0.9
**T stage (n, %)**				
T0	0	11 (22.5)	0	9 (9.5)
Tis	0	8 (16.3)	0	10 (10.5)
T1	4 (8.2)	23 (46.9)	4 (4.4)	36 (37.9)
T2	31 (63.3)	7 (14.3)	50 (52.6)	29 (30.5)
T3	12 (24.5)	0	36 (37.9)	11 (11.6)
T4	2 (4.1)	0	5 (5.3)	0
**N stage (n, %)**				
N0	3 (6.1)	39 (79.6)	9 (9.5)	64 (67.4)
N1	28 (57.2)	6 (12.3)	21 (22.1)	17 (17.9)
N2	12 (24.5)	3 (6.1)	40 (42.1)	8 (8.4)
N3	3 (6.1)	1 (2.0)	18 (19.0)	6 (6.3)

Comparison of the stable disease (SD) status (RESMIN group) and partial response (PR) or complete response (CR) status of microcalcifications after NAC (non-RESMIN group) revealed that the locoregional recurrence rate was significantly higher in the RESMIN group than in the non-RESMIN group; recurrences were found in the axillary lymph area (p = 0.040; Supplementary Fig. 2).

**Figure 1:**
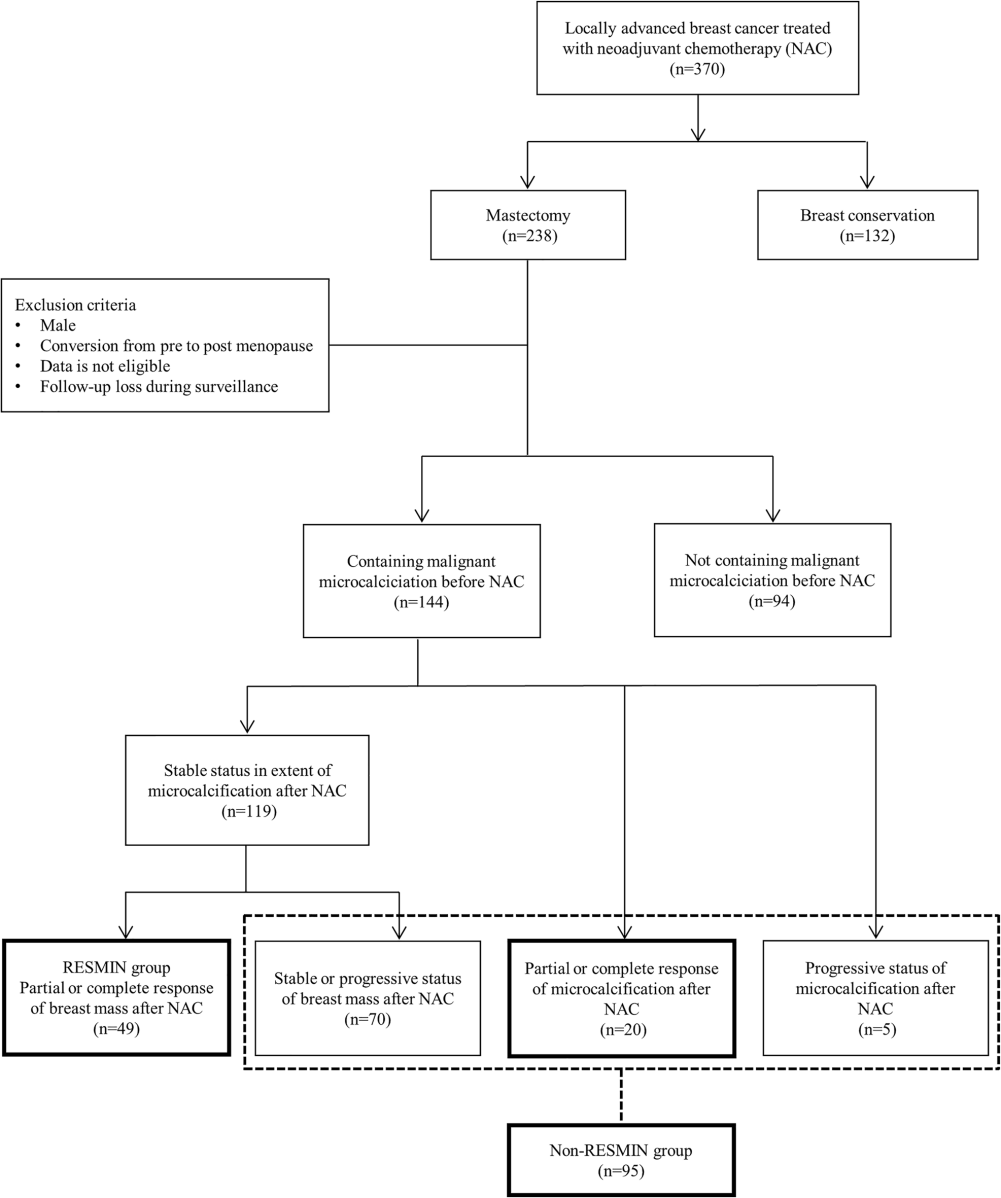
Viable cancer cells in the evaluated breast cancer specimens. The evaluation of viable cancer cells around residual microcalcifications suggested that residual cancers cannot be predicted because of random distribution in specimens; this observation was based on the analysis of a total of five consecutive cases.

In the evaluation of viable cancer cells around residual microcalcifications using five prospectively collected samples, only a small focus (< 0.5 cm) of invasive carcinoma or DCIS was noted (Table 3 and Fig. 1). However, a true pCR (no invasive cells in either in situ lesion) was only found in one patient. Predicting the location of the residual lesions based on preoperative imaging findings was challenging (Supplementary Fig. 3).

**Table 3:** Clinicopathologic factors of patients in a prospective﻿ RESMIN study.

Case no	Age	Clinical stage	Initial mass size (cm)	Extent of initial microcalcification (cm)	Regimen of NAC	Residual tumor burden around microcalcification	Pathologic N stage	Miller–Payne system
#1	63	cT3N1M0, IIIA	7.1	7.1	Docetaxel + Carboplatin + Trastuzumab + Pertuzumab	pCR^*^	ypN0	Grade 5
#2	39	cT3N1M0, IIIA	12.4	12.4	Docetaxel + Carboplatin + Trastuzumab + Pertuzumab	IDC 0.1 cm	ypN0	Grade 4
#3	52	cT3N0M0, IIB	5.0	5.0	Adriamycin + Cyclophosphamide	IDC^#^, 0.3/0.2 cm	ypN0	Grade 4
#4	59	cT3N2M0, IIIC	10.1	10.1	Adriamycin + Cyclophosphamide → Docetaxel + Trastuzumab	pCR (DCIS^†^ only 0.4 cm)	ypN0	Grade 5
#5	47	cT3N0M0, IIB	10.3	4.7	Adriamycin + Cyclophosphamide → Docetaxel	pCR (DCIS only 0.2 cm)	ypN0	Grade 5

^*^pCR, pathologic complete response; ^#^IDC, Invasive ductal carcinoma; ^†^DCIS, Ductal carcinoma in situ.

## Discussion

NAC helps convert inoperable breast cancers to operable ones and guarantees higher rates of breast-conserving surgery in patients with advanced breast cancer^21^. However, only 40–60% cases can be converted from mastectomy to breast-conserving surgery after NAC^22–24^. This suggests that the remaining patients require mastectomy, even if they have received complete NAC for 3–6 months. Not all breast cancers have an excellent response to NAC. In particular, HR-positive breast cancer has a significantly lower response to chemotherapy than HER2-positive breast cancer or TNBC—the aggressive types. Further, the pCR rate is lower in HR-positive breast cancer than in other aggressive breast cancers^25–27^.

Although the size of the main tumor is reduced by NAC, it is difficult to reduce the final extent of surgery if diffuse microcalcifications remain or the tumors show a scattered shrinkage pattern^14,17,28,29^. Several studies have explored whether microcalcifications are related to tumor responses to NAC for breast cancer^16,17,30,31^. However, they have reported inconsistent conclusions, and the results were not significantly related to those differing based on subtypes or those that were related. Some studies have proposed that the surgical range should be determined based on the extent of residual microcalcifications rather than the reduced tumor size^15^. However, to the best of our knowledge, no standard guidelines exist to determine whether it is necessary to completely remove residual microcalcifications after NAC.

This retrospective study compared the oncological outcomes of the RESMIN (tumor size reduced significantly but microcalcifications remained unchanged) and non-RESMIN groups and conducted a simultaneous prospective investigation to assess the location and extent of viable cancer cells around residual microcalcifications.

Although several studies have demonstrated that the aggressive types of breast cancer, including HER2-positive breast cancer and TNBC, show higher pCR rates and tumor responses to NAC, the extent of microcalcifications did not correlate with tumor responses. A recent study found that only 13.9% patients with breast cancer had microcalcifications; all patients exhibited a reduced extent of microcalcification and tumor size. There were significantly more HER2-positive breast cancer and TNBC cases in the RESMIN group than in the non-RESMIN group. This shows a higher tumor response in the aggressive types of breast cancer. The locoregional recurrence rate was also significantly higher in the RESMIN group than in the group showing PR or CR of microcalcifications to NAC. This is consistent with the common characteristics of aggressive cancers—they exhibit rapid regression and relapse. However, breast cancer relapse was not observed in cases of locoregional recurrence; thus, breast cancer recurrence might be considered to have secured oncological safety.

Most breast cancer specimens collected prospectively were predicted to exhibit pCR on preoperative ultrasonography and breast magnetic resonance imaging, regardless of the residual microcalcification status. However, only one patient completely lacked viable cancer cells, including invasive and *in situ* lesions. In the other four cases, invasive or *in situ* lesions remained, even if they were small. Furthermore, predicting the location of residual cancer in the specimen using mammography was challenging because the lesions were randomly distributed around microcalcifications.

Taken together, in cases of tumor regression without any change in the extent of microcalcifications, the extent of surgery must be determined based on residual microcalcifications. Complete removal of microcalcifications may be necessary to confirm the actual pCR, and complete removal of residual cancer may help reduce the local recurrence rate. Furthermore, malignant microcalcifications that remain even after NAC contain larger hydroxyapatite particles, which upregulate MMP-1 and promote the aggressiveness of breast cancer by decreasing the elasticity of the extracellular matrix^18–20^.

To clearly conclude on the oncologic necessity of complete removal of residual microcalcification, further studies with a larger sample size in a multicenter setting are needed to collect scientific evidence. In our study, only five cases were evaluated to confirm the residual invasive carcinoma after NAC in cases of residual microcalcifications. Further, the invasive focus could have been missed if the microcalcification was not detection on mammography. However, this is the first study to examine the relationship between residual microcalcification and residual breast cancer in the entire breast cancer specimen. This study provides a scientific basis for the necessity for complete removal of microcalcification.

In conclusion, since residual cancers remained in most cases in the RESMIN group, reduction of the surgical extent seems challenging. In addition, because the location of the residual cancer around microcalcifications could not be predicted, complete removal of residual microcalcifications is necessary. For oncologic safety, residual microcalcifications after NAC should be completely removed, and the whole specimen should be assessed to determine the residual tumor burden, even with a very small focus.

## Methods

Between 2011 and 2017, the clinical data of 370 patients with locally advanced breast cancer who had received NAC before undergoing surgery and additional treatments at Kyungpook National University Chilgok Hospital (Daegu, Republic of Korea), were reviewed. This study was conducted in accordance with the ethical standards of the Institutional Review Board of Kyungpook National University Chilgok Hospital. The experimental protocol was also approved by the Institutional Review Board of Kyungpook National University Chilgok Hospital (KNUCH 2021-07-044-003), and all experiments were performed in accordance with the relevant guidelines and regulations.

### Retrospective study

**Figure 2:**
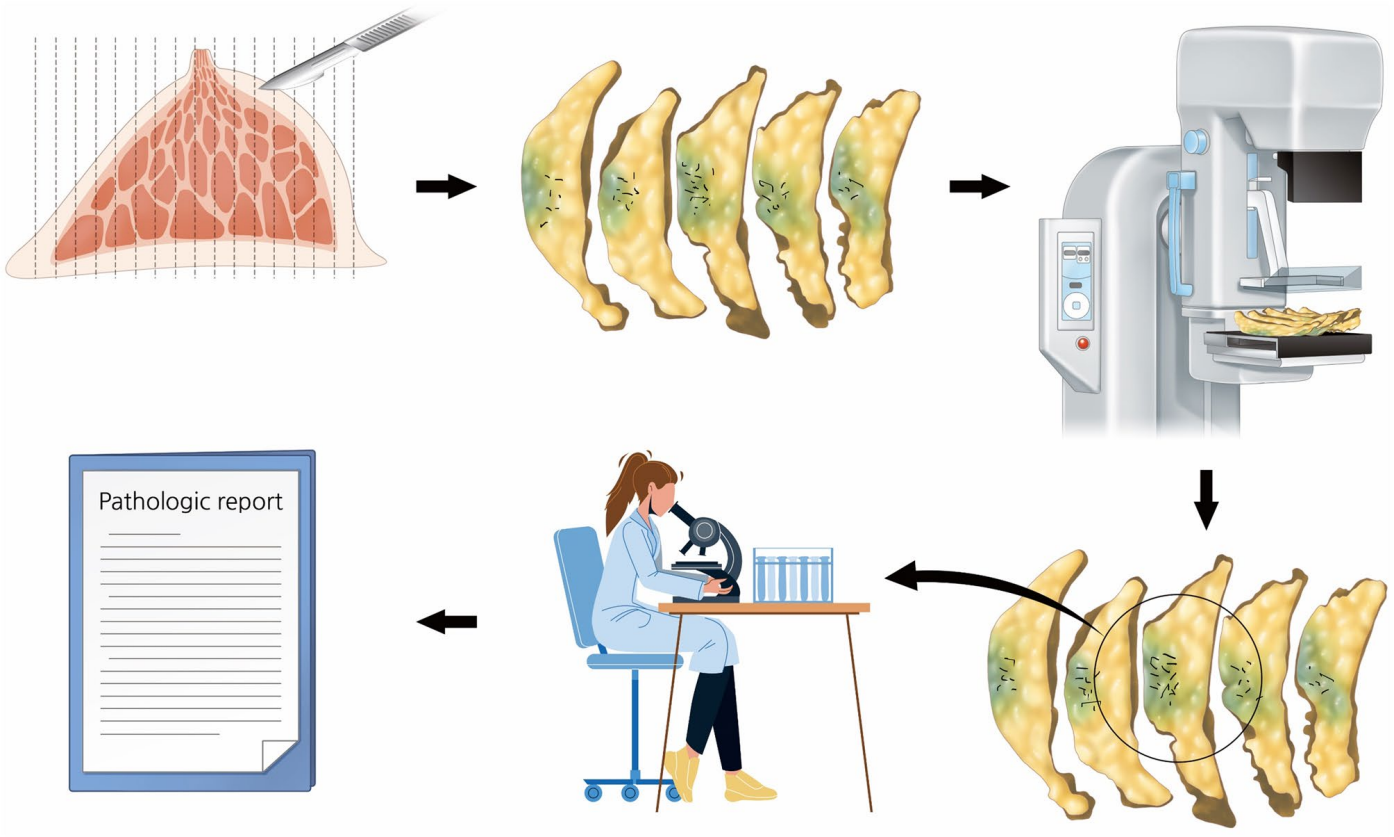
Classification and inclusion criteria of the reduced mass with no change in residual microcalcification group based on tumor status and microcalcifications before and after neoadjuvant chemotherapy.

Among 370 patients, 238 patients underwent mastectomy after NAC, and 144 had breast cancer with malignant microcalcifications around the tumor. Patients with advanced breast cancer and malignant microcalcifications, where diffuse microcalcifications initially showed SD but primary tumors showed partial PR or CR to NAC, were grouped into the RESMIN group (Supplementary Fig. 4). The RESMIN and non-RESMIN groups included 49 and 95 patients, respectively (Fig. 2).

Treatment response to NAC was assessed according to the RECIST 1.1 criteria^32^. CR was defined as no evidence of tumor on physical examination using radiological images. PR was defined as a reduction in the diameter of the largest tumor by > 30% on radiological images. SD was defined as an increase in the diameter of the largest tumor by < 20%. Progressive disease (PD) was defined as an increase in the diameter of the largest tumor by ≥ 20%. Thus, the treatment response of malignant microcalcifications to NAC was assessed as CR, PR, SD, or PD based on the RECIST criteria.

Changes in the size of the tumor, extent of malignant microcalcifications, and tumor burden of axillary lymph nodes were monitored during and after NAC. The mean follow-up period was > 7 years, and all breast cancer-specific events were recorded. The molecular subtypes of breast cancer were classified based on the results of immunohistochemical staining of the biopsy samples before initial treatment. The Ki67 index was considered high when > 15% tumor cells showed nuclear immunoreactivity. The ASCO/CAP 2016 guidelines were followed for the histopathological examination of four biomarkers.

The histopathological regression of breast cancer was assessed using the Miller–Payne grading scale based on the overall cellularity in microcalcification removal and mastectomy samples compared to that in the pretreatment biopsy samples^33^. Grade 5 indicated that no malignant cells were identifiable in sections obtained from the tumor site, with only vascular fibroelastotic stroma remaining (which often contained macrophages) and possible presence of DCIS (pCR).

### Prospective study

**Figure 3:**
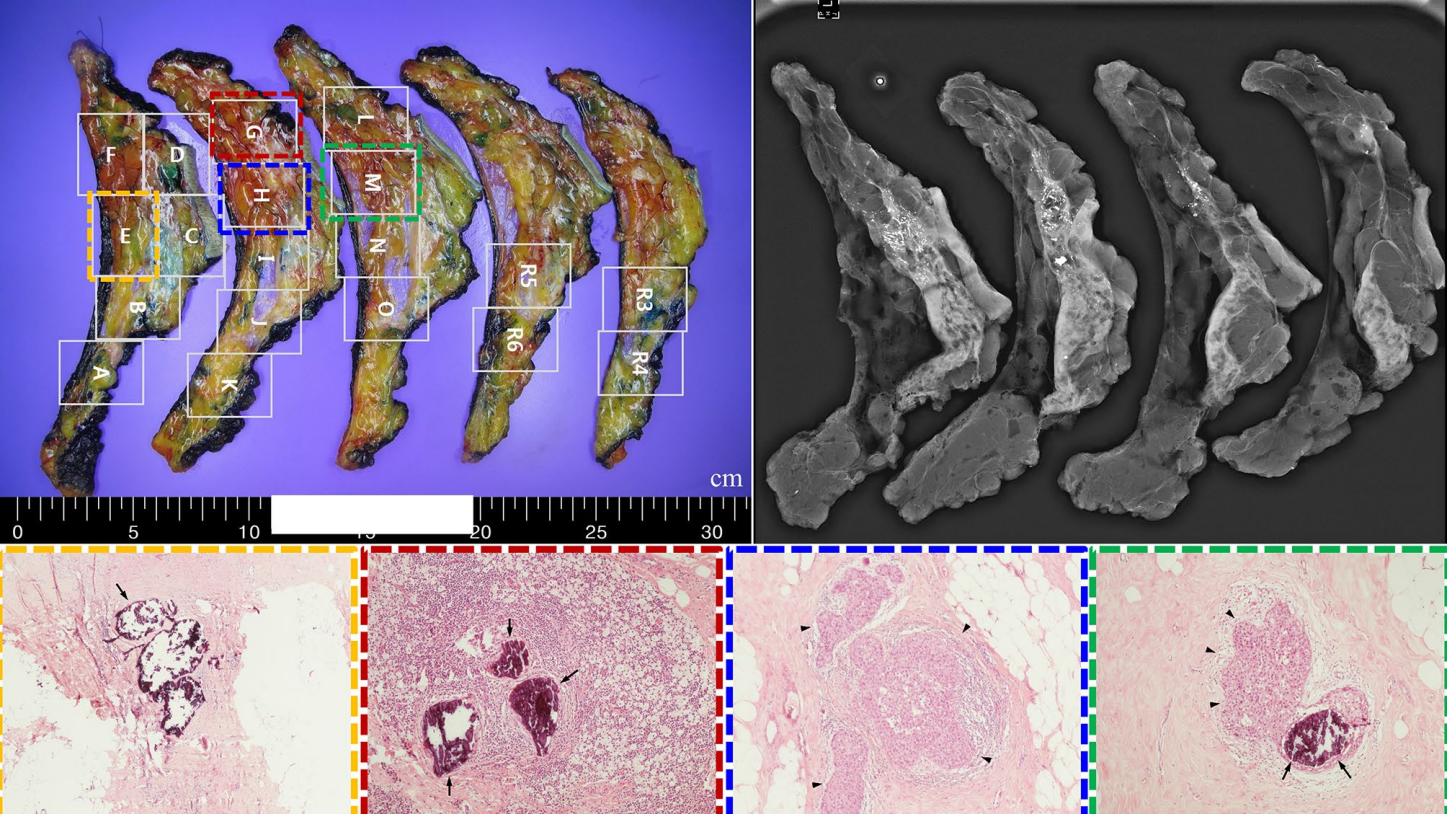
Process of a prospective study using samples from the reduced mass with no change in residual microcalcification group. After obtaining the specimen, it was sliced into 15–20 pieces; specimen mammography is then performed with pieces of specimen arranged serially. Next, the specimens are sent to pathologists for the assessment of viable cancer cells, and final pathologic results were reported.

Five consecutive breast cancer specimens from the RESMIN group were prospectively used to assess the viable cancer cells around residual microcalcifications. The obtained breast cancer specimens were sliced in 1-cm intervals by a pathologist, and specimen mammography was performed with multiple pieces of the entire specimen arranged serially. Subsequently, the pathologist confirmed the viability of cancer cells in residual microcalcifications detected using mammography in the evaluated specimen (Fig. 3).

### Statistical analysis

All statistical analyses were performed using SPSS (version 25.0, SPSS, Chicago, IL, USA). Categorical variables were analyzed using chi-square test in univariate analysis, and the statistical significance was set at p < 0.05.

### Institutional review board statement

This study was approved by the Institutional Review Board Committee of Kyungpook National University Chilgok Hospital, Daegu, Republic of Korea (KNUCH 2021-07-044-003). In addition, the specific inclusion and exclusion criteria were defined in the approved Institutional Review Board protocol.

### Informed consent

Informed consent was obtained from all participants involved in this study.

## Supplementary Information

Supplementary Information 1.

Supplementary Information 2.

Supplementary Information 3.

Supplementary Information 4.

Supplementary Information 5.

Supplementary Information 6.

## Data Availability

The datasets generated and/or analyzed during the current study are not publicly available. However, they are available from the corresponding author upon reasonable request.
