# Supplementary figures and images for: Oncologic necessity for the complete removal of residual microcalcifications after neoadjuvant chemotherapy for breast cancer
Source: Sci Rep. 2022 Dec 13;12:21535. doi: 10.1038/s41598-022-24757-7 (PMC9748126; doi:10.1038/s41598-022-24757-7)

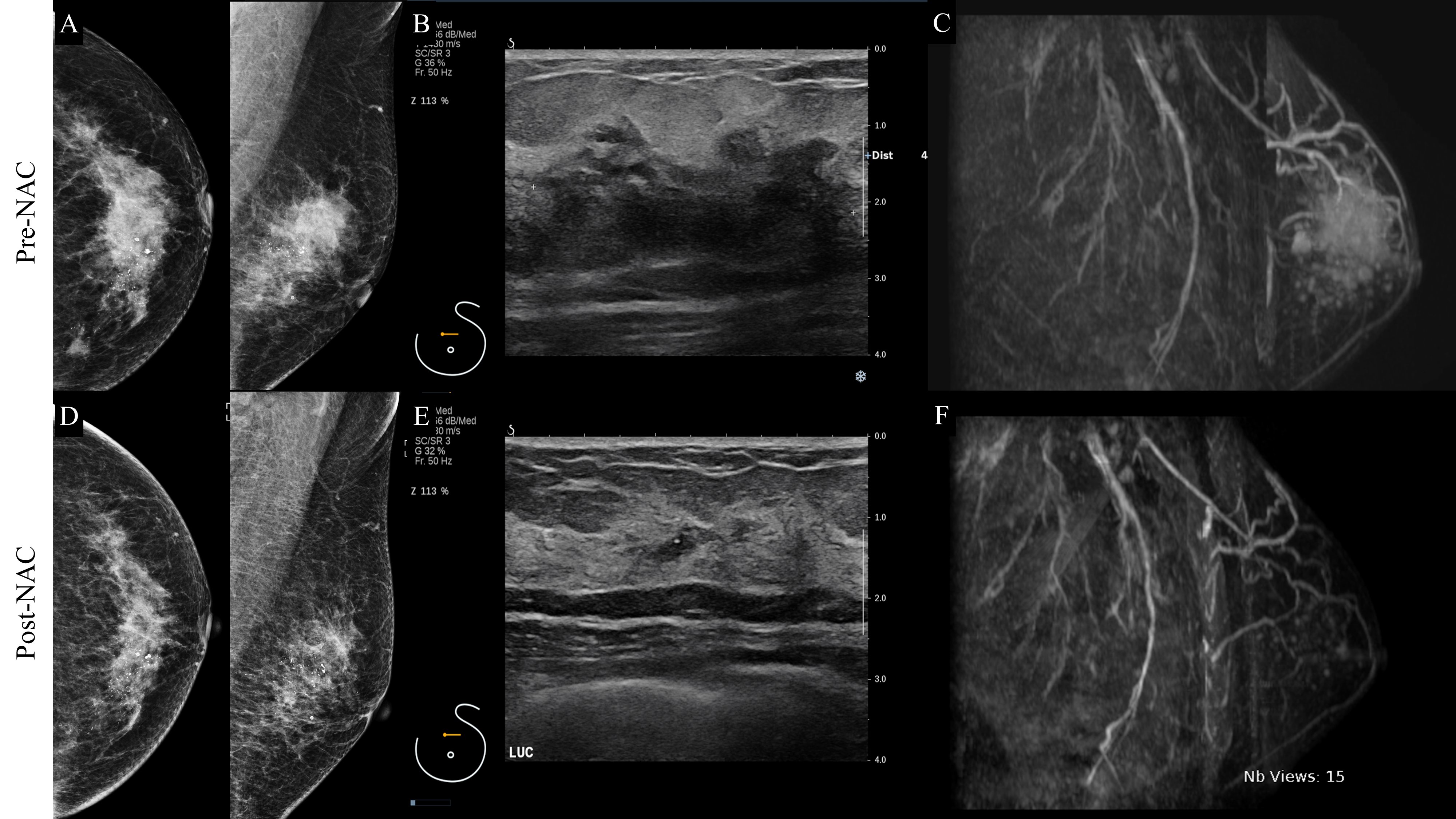

Supplement: Supplementary file 1 — Supplementary Information 1. [file 41598_2022_24757_MOESM1_ESM.tif]

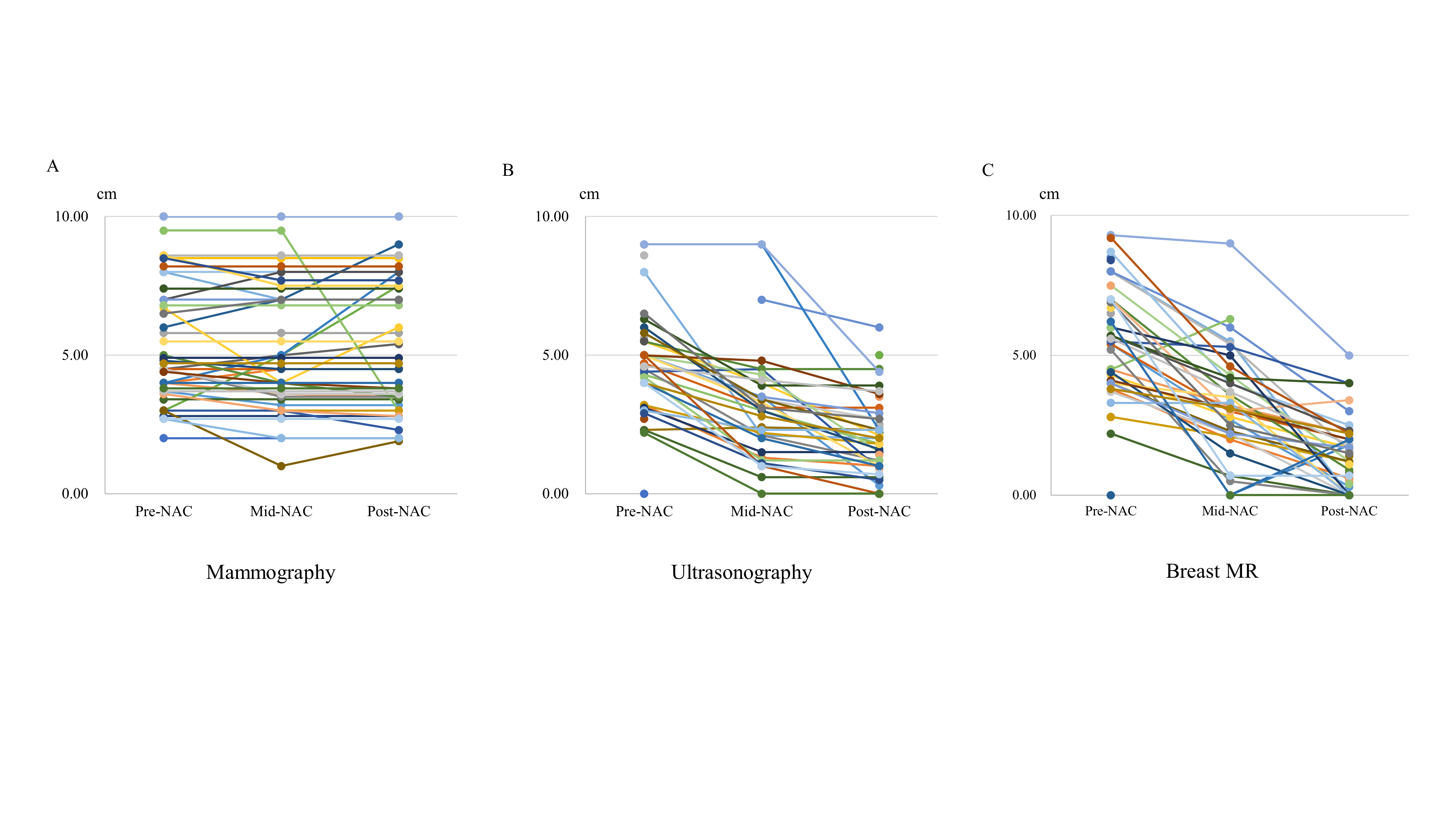

Supplement: Supplementary file 2 — Supplementary Information 2. [file 41598_2022_24757_MOESM2_ESM.tif]

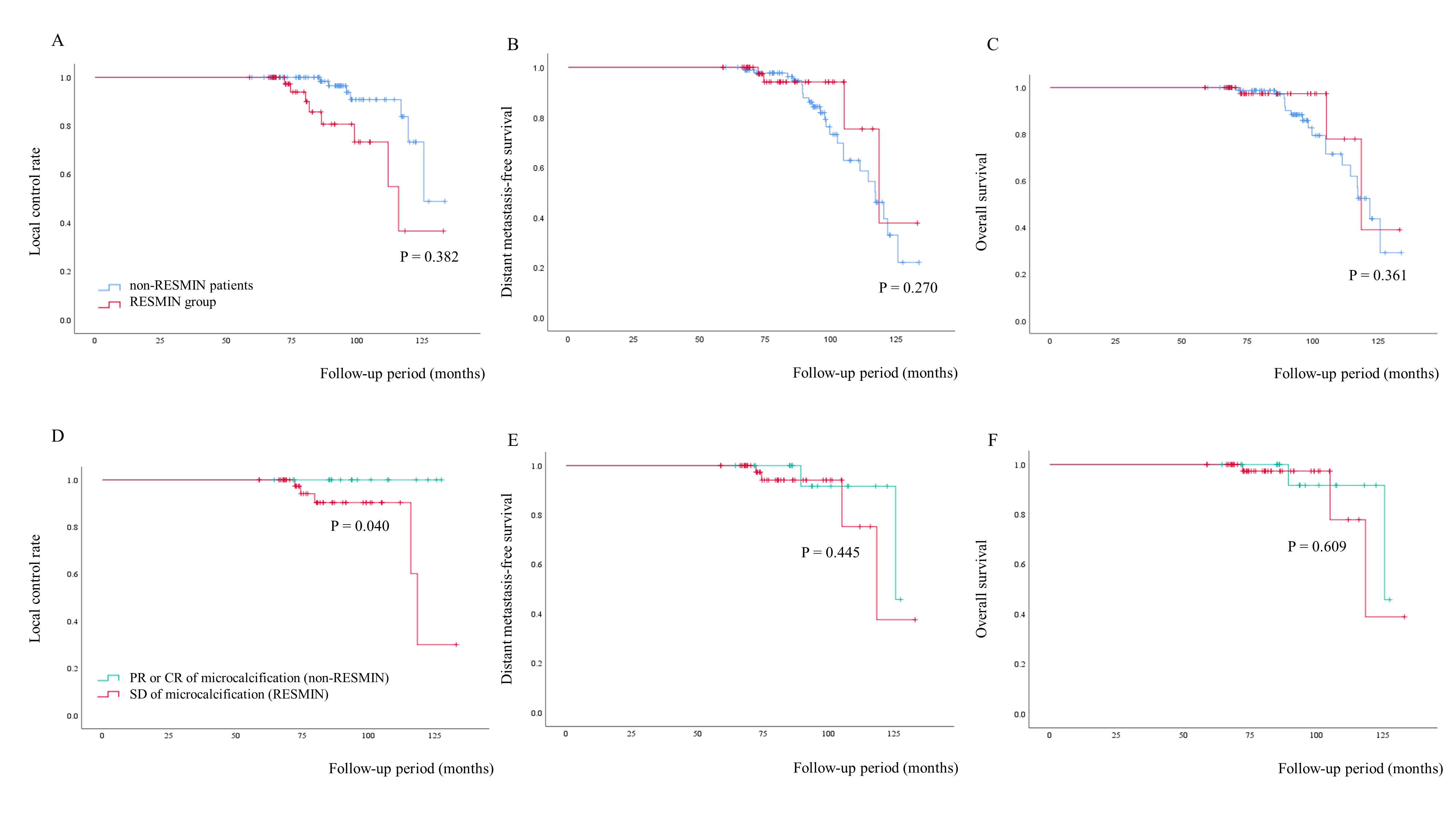

Supplement: Supplementary file 4 — Supplementary Information 4. [file 41598_2022_24757_MOESM4_ESM.tif]

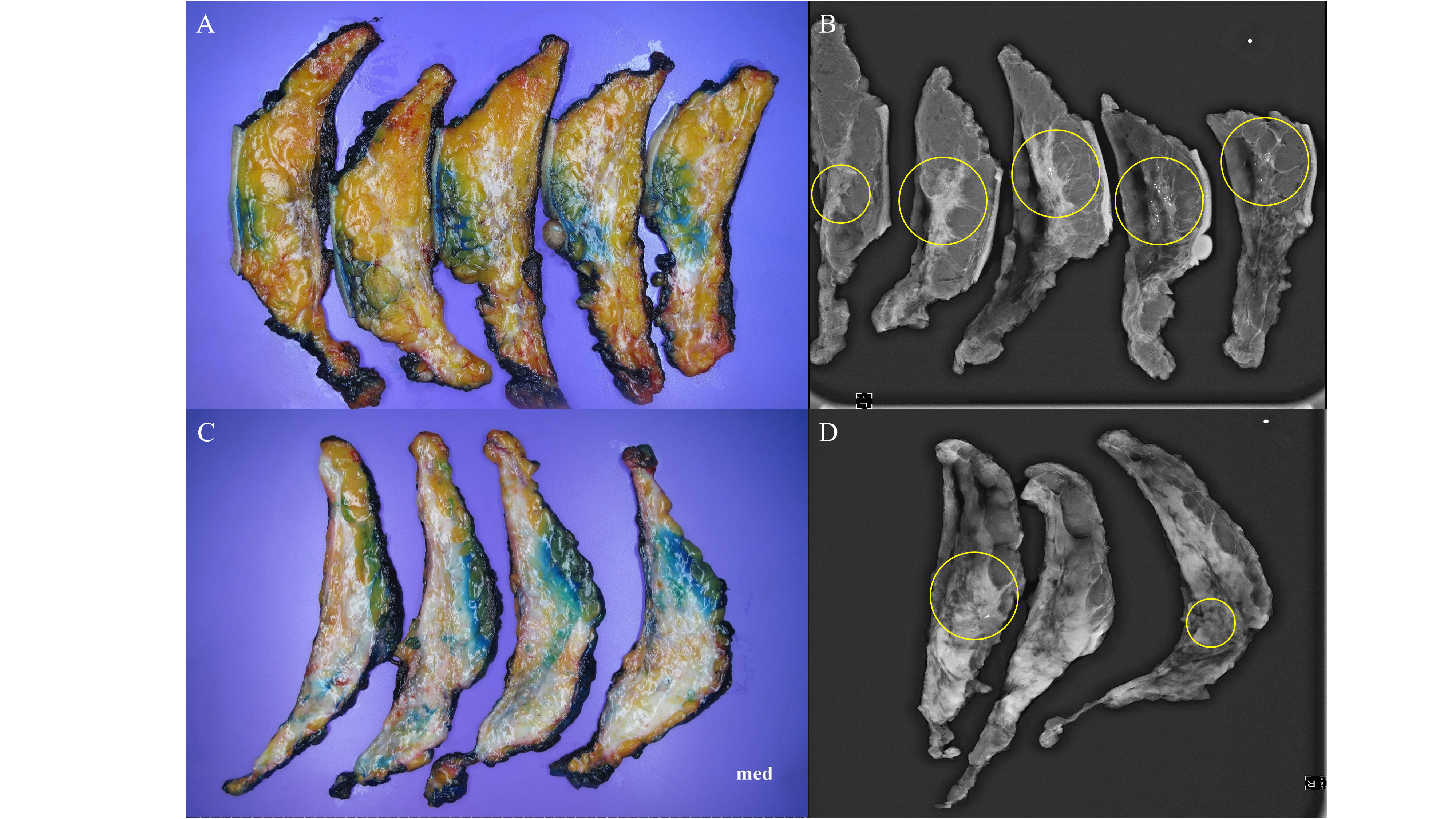

Supplement: Supplementary file 5 — Supplementary Information 5. [file 41598_2022_24757_MOESM5_ESM.tif]
