# Supplementary material for: Oncologic necessity for the complete removal of residual microcalcifications after neoadjuvant chemotherapy for breast cancer
Source: Sci Rep. 2022 Dec 13;12:21535. doi: 10.1038/s41598-022-24757-7 (PMC9748126; doi:10.1038/s41598-022-24757-7)
Supplement: Supplementary file 6 — Supplementary Information 6. [file 41598_2022_24757_MOESM6_ESM.doc]

Supplementary Figure 1. Oncologic outcomes of the RESMIN and non-RESMIN groups. (A,B,C) Locoregional recurrence, distant metastasis, and overall survival of the two groups showed no statistical differences (p=0.382, 0.270, and 0.361, respectively). (D,E,F) Locoregional recurrence was significantly higher in the SD status of microcalcification (RESMIN group) than the PR or CR (non-RESMIN group; p=0.040). However, the recurrences were all axillary and not breast recurrence. Distant metastasis and overall survival showed no significant differences between two groups.

Supplementary Figure 2. Changes in the extent of tumor or microcalcification at pre, mid-term and post-neoadjuvant chemotherapy (NAC) periods. Although the microcalcifications showed no change in most patients (A), the extent of tumor on breast ultrasonography (B) and MR (C) was significantly reduced.

Supplementary Figure 3. Other prospective RESMIN cases. (A,C) Sliced specimens of breast cancer specimen with diffuse microcalcifications. The margins were stained with various stains; a blue dye (indigo carmine) was used to stain the parenchyma for sentinel lymph node biopsy. (B,D) Diffuse microcalcifications were identified on mammography of the specimen in serial sections.

Supplementary Figure 4. Definition of RESMIN as a case of tumor regression and no change in residual microcalcification after neoadjuvant chemotherapy (NAC). A tumor of approximately 5 cm was detected on mammography as a hyperdense mass containing diffuse microcalcification (A), ultrasonography as a hypoechoic mass with lobulated margins (B), and breast MRI as a huge enhancing nodule of up to 7.8 cm that occupied almost the entire parenchyma (C). Although the mass almost completely disappeared after NAC, as noted on ultrasonography (E) and breast MR (F), diffuse microcalcifications were still noted on mammography, without any significant changes (D).

Supplementary Table 1. Changes of tumor status as detected by mammography, ultrasonography, and breast MRI according to neoadjuvant chemotherapy

|  |  | RESMIN (n=49) | non-RESMIN (n=95) |
| --- | --- | --- | --- |
| Extent of microcalcification (mean±SD, cm) | Pre-NAC | 5.4±0.7 | 5.2±0.5 |
|  | Mid-NAC | 5.3±0.4 | 4.9±0.2 |
|  | Post-NAC | 5.1±0.4 | 4.8±0.7 |
| Extent of tumor size (mean±SD, cm) | Pre-NAC | 5.8±1.1 | 5.6±2.5 |
|  | Mid-NAC | 3.2±1.4 | 4.4±1.5 |
|  | Post-NAC | 1.3±0.3 | 3.8±0.9 |
| Suspicious axillary lymph nodes (n, %) | Pre-NAC | 46 (93.9) | 88 (92.6) |
|  | Mid-NAC | 29 (59.2) | 67 (70.5) |
|  | Post-NAC | 1 (2.0) | 35 (36.9) |
| Decreased tumor size (n, %) |  | 49 (100.0) | 63 (66.3) |
| Decrease in extent of microcalcification (n, %) |  | 0 | 20 (21.1) |
| Decrease in suspicious axillary lymph nodes (n, %) |  | 40 (81.6) | 51 (53.7) |

Supplementary Table 2. Comparison of the clinicopathologic factors between the stable status and partial or complete response of microcalcification after neoadjuvant chemotherapy

| Characteristics | SD of microcalcification after NAC (n=49) | PR or CR of microcalcification after NAC (n=20) | p-value |
| --- | --- | --- | --- |
| Age at diagnosis (years), mean ± SD | 46.6±6.4 | 48.7±7.1 | 0.503 |
| Pathologic complete response (pCR), n (%) | 19 (38.8) | 5 (25.0) | 0.015 |
| Estrogen receptor (positive), n (%) | 33 (67.4) | 15 (75.0) | 0.186 |
| Progesterone receptor (positive), n (%) | 24 (49.0) | 10 (50.0) | 0.929 |
| HER2 gene (positive), n (%) | 26 (53.1) | 8 (40.0) | 0.256 |
| Triple-negative breast cancer, n (%) | 5 (10.2) | 1 (5.0) | 0.157 |
| High Ki67 index, n (%) | 34 (69.4) | 11 (55.0) | 0.413 |
| Target therapy, n (%) | 26 (53.1) | 8 (40.0) | 0.256 |
| Adjuvant radiotherapy, n (%) | 44 (89.8) | 15 (75.0) | 0.003 |
| Adjuvant hormonal therapy, n (%) | 34 (69.4) | 17 (85.0) | 0.003 |
| Follow-up period (months), mean ± SD | 84.8 ± 52.4 | 94.6 ± 44.4 | 0.559 |
| Locoregional recurrence (n, %) | 5 (10.2) | 0 | 0.040 |
| Breast | 0 | 0 |  |
| Ipsilateral axilla or supraclavicular lymph node, n (%) | 5 (10.2) | 0 |  |
| Distant metastasis, n (%) | 4 (8.2) | 2 (10.0) | 0.445 |
| Death, n (%) | 3 (6.1) | 2 (10.0) | 0.609 |
